# Antibacterial and Antifungal Activity of ZnO Containing Glasses

**DOI:** 10.1371/journal.pone.0132709

**Published:** 2015-07-31

**Authors:** Leticia Esteban-Tejeda, Catuxa Prado, Belén Cabal, Jesús Sanz, Ramón Torrecillas, José Serafín Moya

**Affiliations:** 1 Department of Biomaterials and Bioinspired Materials, Materials Science Institute of Madrid, (ICMM), Spanish National Research Council (CSIC), Cantoblanco, Madrid, 28049, Spain; 2 Nanomaterials and Nanotechnology Research Center, Spanish National Research Council-Universidad de Oviedo-Principado de Asturias, (CINN-CSIC-UNIOVI-PA), Avenida de la Vega, 4–6, 33940, El Entrego, Asturias, Spain; 3 Moscow State University of Technology STANKIN, Vadkovskij per. 1, Moscow Oblast, 101472, Moscow, Russian Federation; University of California, Merced, UNITED STATES

## Abstract

A new family of non-toxic biocides based on low melting point (1250°C) transparent glasses with high content of ZnO (15–40wt%) belonging to the miscibility region of the B_2_O_3_-SiO_2_-Na_2_O-ZnO system has been developed. These glasses have shown an excellent biocide activity (logarithmic reduction >3) against Gram- (*E*. *coli*), Gram+ (*S*. *aureus*) and yeast (*C*. *krusei*); they are chemically stable in different media (distilled water, sea-like water, LB and DMEN media) as well as biocompatible. The cytotoxicity was evaluated by the Neutral Red Uptake using NIH-3T3 (mouse embryonic fibroblast cells) and the cell viability was >80%. These new glasses can be considered in several and important applications in the field of inorganic non-toxic biocide agents such as medical implants, surgical equipment, protective apparels in hospitals, water purifications systems, food packaging, food storages or textiles.

## Introduction

Nowadays, there is great concern regarding diseases, infections, corrosion and biocontamination caused by microorganisms [1–7]. For instance, the Target 10 of the UN Millennium Development goals is to reduce by half the proportion of people without sustainable access to safe drinking water by 2015 [8]. There is an urgent global need to develop new antimicrobial agents to prevent the adverse effects of microorganisms due to the fact that traditional antimicrobial compounds are contaminant, toxic or have effects on human health. The World Health Organization has asserted the need to study the effects of the chemicals and traditional biocides on living organisms and humans. For instance, one of the most important effects is antimicrobial resistance. This is a major global public health concern and because of that the European Commission is committed focusing on this issue [9]. Besides, the antimicrobial agents are usually organic compounds that have limited applications due to their low heat resistance, high decomposability and short life [10].In this regard, the inorganic biocides have acquired importance, but they are commonly based on nanoparticles which have effects on environment and human health [11–15]. Therefore, the development of new inorganic biocides without antimicrobial resistance or adverse effects on living organisms, humans or environment is absolutely essential. In this context, the proposed ZnO-reach glasses are promising candidates to be used as antimicrobial agents.

It is widely reported that zinc oxide has antimicrobial properties without the toxicity and environmental effects of other biocide agents like silver, copper or the nanoparticles [16–22]. It has been reported that ZnO nanoparticles show selectivity to prokaryotic and eukaryotic systems being more toxic for the prokaryotic cells [23]. Thus, both gram negative and gram positive bacteria are killed with lower ZnO concentrations than human T cells [24]. Hanley et al. have stated that ZnO nanoparticles kill preferentially the cancerous cells so these nanoparticles can be used in the field of nanomedicine [24]. Therefore, ZnO containing glasses are greener than ZnO nanoparticles and susceptible to have biocide activity because in this case, the possible toxicity of the ZnO in a liquid media can be solved by using a glassy matrix. This is the best option, because the Zn^2+^ forms part of the glass network, so it is possible to design the most appropriate glass composition for a specific application due to the fact that the release of ZnO to the media can be controlled by the kinetic dissolution of the glassy matrix. Then the glass can be considered as a dispenser of ZnO avoiding the health and environmental problems caused if the ZnO is in direct contact with the liquid media as it is the case of ZnO nanoparticles [25]. Because of this, the controlled releasing of ZnO is highly important, in order to dose the ZnO and keep the biocide activity for a long time without toxicity.

However, as far as it is known, very few exploratory studies focused on complex phosphate glasses containing ZnO have been reported [26,27] and furthermore ZnO containing glasses have not been fabricated to be used as biocides, until now. Traditionally, ZnO has been a component in ceramic frites and glazes because of their excellent properties like chemical durability and mechanical stability [28–30]. These glasses have been employed for enameling because of their high refraction index, although they have been used less than the PbO glasses due to economic reasons. The role of ZnO is similar to that of PbO in glasses; it is an intermediate oxide that can act as former or modifier oxide, but without the toxicity of the PbO. Zinc oxide is also a magnificent stabilizer and improves the final glass properties. However, these glasses have limited the ZnO weight percentage to less than half of the sum of all alkaline oxides that is ≤10wt.% [31,32].Up to now, very few studies on glasses belonging to the B_2_O_3_-SiO_2_-Na_2_O-ZnO system and with a high content in ZnO (>10wt.%) have been reported in the literature [33,34].

The aim of this work is to fabricate non-toxic and stable glasses with a high content of ZnO (15–40wt.%) in the miscibility volume of the B_2_O_3_-SiO_2_-Na_2_O-ZnO system, and with a high biocide activity. The chemical stability, the local structure and the biocide activity of this series of glasses in the mentioned range of compositions are also reported. These glasses are very versatile and they can be used also as antifouling agents in a wide range of applications such as medical implants, surgical equipment, protective apparels in hospitals, water purifications systems, food packaging, food storages or textiles [2,35–39].

## Materials and Methods

### 2.1 Preparation of Glasses

Four glasses; ZnO15, ZnO25, ZnO35 and ZnO40 (d_50_ = 6.3±0.1μm) belonging to the B_2_O_3_-SiO_2_-Na_2_O-ZnO system and with the following ZnO content (wt%) 15, 25, 35 and 40% (Table 1) respectively, were prepared by fusion of mixtures of Na_2_CO_3_, SiO_2_, B_2_O_3_, ZnO and Al_2_O_3_ in Pt crucibles at 1250°C for 1h in an electric furnace. After melting, the samples were quenched by dipping the crucible into water, dried, grinded in an agate mortar and sieved down to 30μm. The average particle size of the obtained powder were found to be d_50_ = 6.3±0.1μm. The glass formulation was designed in the miscibility area according to the equilibrium diagram studied by Taylor et al. [34]. The Al_2_O_3_ was added as stabilizer. Window glass like powder (d_50_ = 2.9±0.1μm) of composition (wt.%): 70.2 SiO_2_, 15.8 Na_2_O, 7.1 CaO, 3.2 MgO 1.06, B_2_O_3_ and 0.05 K_2_O was also fabricated to be used as control in antimicrobial tests.

**Table 1:** Chemical composition (wt%) of the glasses.

	ZnO15	ZnO25	ZnO35	ZnO40
**SiO**_**2**_	25.53	22.53	19.29	18.02
**B**_**2**_ **O**_**3**_	45.33	40.00	34.24	32.00
**Na**_**2**_ **O**	7.35	6.48	5.55	5.19
**Al**_**2**_ **O**_**3**_	6.79	5.99	5.13	4.79
**ZnO**	15.00	25.00	34.73	40.00

### 2.2 Characterization of Glasses

The ZnO-glasses were fully characterized by: (i) Nuclear Magnetic Resonance (NMR) using the single π/2 pulse magic-angle-spinning technique in a Nuclear Magnetic Resonance Bruker AVANCE 400 spectrometer (^11^B, ^23^Na, ^27^Al, ^29^Si, ^27^Si signals); (ii) Particle size was determined using a laser Malvern Mastersizer model 2.18; (iii) X-Ray diffraction (XRD) was carried out using a Bruker D8 diffractometer with CuKα radiation working at 40kV and 30mA in a step-scanning mode from 10 to 70° with a step width of 0.0288 and a step time of 2.5s; (iv) Transmission electron microscopy (TEM) was performed using a JEOL JEM 2100 at 200kV (v) Differential thermal analysis (DTA) was carried out using a TA Instruments model Q600; (vi) Hot stage microscopy was carried out in Hesse Instruments on alumina support; (vii) The thermal expansion coefficients were determined in a BÄHR THERMOANALYSE model DIL using glass samples bars with ~2cm length in the temperature interval ranging from RT to 500°C.

### 2.3 Biocide Activity Tests

*Escherichia coli* (ATCC 25922), *Staphylococcus aureus* (ATCC 29213) and *Candida krusei* (ATCC 14243) strains were selected to evaluate the antimicrobial activity of the glass powders, as representative examples of Gram-negative, Gram-positive and yeast, respectively.

### 2.3.1 Antimicrobial Activity

The antimicrobial test was carried out as previously described [40]. The inoculum was prepared from a fresh 6 hour shake culture of the corresponding microorganism in a sterile nutrient broth (Luria Bertani, LB, for bacteria and Yeast Extract Dextrose, YEPD, for *C*. *krusei*). The LB medium is composed (wt%) of: 1% tryptone; 0.5% yeast extract; 1% NaCl and 1.5% agar. The composition of the YEPD medium (wt%) is as follows: 1% yeast extract; 2% peptone and 2% glucose. The cultures were diluted into fresh media until a concentration ⋍1x10^6^ CFU/ml. This solution was the working microbial solution. Aqueous suspensions of the glass powders were prepared (200mg/ml) and sterilized (autoclave, 121°C, 20min). Finally, 925μl of the working microbial solution was mixed with 25μl of the glass powder suspension (final glass concentration: 5mg/ml) and 50μl of distilled water was added to obtain a final volume of 1ml. A control was prepared by adding 75μl of distilled water. The effect of the corresponding glass without ZnO was also investigated as negative control. Glass powders were shaken in microbial suspension for 24h to ensure good contact between the microorganisms and glass particles. After 24h, the number of viable microorganisms in each suspension was determined by dilution plating.

### 2.3.2 Minimum Inhibitory Concentration (MIC) Determination

*E coli* strain was grown overnight on LB medium at 37°C before being used. The antimicrobial activity on ZnO-containing glasses was examined using the standard broth dilution method. The MIC was determined in LB broth using serial two-fold dilutions of ZnO glasses in concentrations ranging from 15mg/ml to 0.5mg/ml. The initial bacterial inoculums for each replica were: 1.65x10^7^ (replica 1); 4.61x10^5^ (replica 2) and 1.34x10^7^ (replica 3).

The MIC was determined as the lowest concentration that inhibits the growth of the microorganisms by curve fitting data (exponential fitting). The MIC was performed by triplicate.

#### 2.3.3 Antibiograms

A zone of inhibition test (antibiogram) was also performed to determine the antimicrobial activity of ZnO-glasses. This kind of assay is used clinically to measure antibiotic resistance (Kirby-Bauer Test) [41] and industrially to test the ability of products to inhibit microbial growth **(**ISO 20645) [42]. Briefly, a suspension of *E*. *coli* was spread evenly over a nutrient agar plate using a sterile swab and allowed to dry for 5 minutes. Then, 5 wells were punched in the agar with a 3mm punch and filled with 40μl of glass suspensions (200mg/ml in water). The agar plate was incubated for 24h at 37°C. Sterile water was used as a control. Following incubation plate was visually examined for zones of clearing.

#### 2.3.4 Procedure for Determining Presence of Antimicrobial Leaching

A suspension of ZnO35 in LB medium was prepared at 5mg/ml and incubated for 48h at 37°C under agitation. After that glass particles were removed by centrifugation and filtration and supernatant (ZnO-conditioned medium) was inoculated with *E*. *coli* (final concentration1x10^6^ CFU/ml). The bacteria were incubated at 37°C for 24h.

### 2.4 Cytotoxicity (Biocompatibility)

The potential cytotoxic effect of our glasses on eukaryotic cells was determined by using the Neutral Red Uptake (NRU) assay. In this test the uptake of NR into the lysosomes/endosomes and vacuoles of living cells is used as a quantitative indication of viability [43]. The followed procedure was based on the extract dilution method described in ISO 10993-part 5 standard for biomaterial and medical device testing [44].

In order to obtain the glasses extracts, 5mg from each glass was immersed in 1ml of Dulbecco’s Modified Eagle Medium (Gibco DMEM) supplemented with a 10% of new born calf serum (Gibco) (complete medium) for 24h at 37°C. After 24h the medium was centrifuged and filtered (0.22μm filter) to eliminate the glass particles.

NIH-3T3 cells (mouse embryonic fibroblast cells) were cultured in 96 well-plates (1.0 × 10^4^ cells/well) for 24h for formation of monolayers. After achievement of confluence the culture medium was replaced with the media that contained the glass extract or fresh complete medium (blank). After 24h cells were washed and incubated with the neutral red solution (Neutral red solution 0.1%, Scharlau) for 3h. Then, the cells were washed again and NR was desorbed by adding a mixture of ethanol and acetic acid. The amount of NR extracted from the cells was measured at 540nm. The percentage of viable cells in each culture condition was calculated as: % viable cells = 100 × Abs_540_ (extract)/Abs_540_ (blank)

NIH-3T3 cell line (ATCC CRL-1658, LGC Standars) was kindly donated by the Scientific-Technical Services from the University of Oviedo.

### 2.5 Chemical Durability

The chemical durability for each glass was measured by the Product Consistency Test (PCT). This method is described by the ASTM Designation C 1289–97 rule [45]. The results were normalized to facilitate the comparison between elements (Eq 1).

$$NR_{e}=\frac{\left(C_{eg}-B_{e}\right)}{f_{e}\cdot m_{g}\cdot \left(SA_{g}/V_{g}\right)}$$ (1)

Where:

NR_e_: Normalized release of element *e* from glass *g*

C_eg_: Concentration of element *e* in leachate from glass *g* (ppm)

B_e_: Concentration of element *e* in leachate from blank (detection limit for all analyses)

V_g_: volume of leachant containing glass *g* (l)

f_e_: average mass fraction of element *e* in the glass *g*

m_g_: original mass of glass *g* (g)

SA_g_: surface area per unit mass of glass *g*. The surface area per unit mass is found by Eq (2). This calculation assumes a mean diameter of spherical particles with a grain density of 2.5g/cm^3^. The final normalized release values are expressed in g/m^2^ (grams of element released per square meter of glass surface area).

$$SA=\frac{4r^{2}}{4/3r^{3}\cdot 2.5g/cm^{3}}$$ (2)

### 2.6 Lixiviation of Zn^2+^

The release of Zn^2+^ from the zinc containing glasses was evaluated in four different media: (i) distilled water, (ii) water containing 3wt% NaCl (sea-like water), (iii) LB medium and (iv) DMEN medium. In the first case, an aqueous suspension in distilled water (5mg/mL) of each corresponding ZnO containing glass (ZnO15, ZnO25, ZnO35 and ZnO40) was kept under agitation during 14 days at 37°C. In the others cases (ii, iii and iv) the release of Zn^2+^ from the ZnO15 and ZnO35 glasses was determined after 24h at 37°C in the corresponding medium. The analysis of zinc was performed by Inductively Coupled Plasma (ICP) using a Perkin Elmer optical emission spectrometer model optima 2100 DV. The solutions analyzed by ICP were previously centrifuged and filtered in order to obtain an aqueous solution containing the metal ions without glass particles.

## Results and Discussion

### 3.1 Glass Formation and Physical Properties

**Fig 1:**
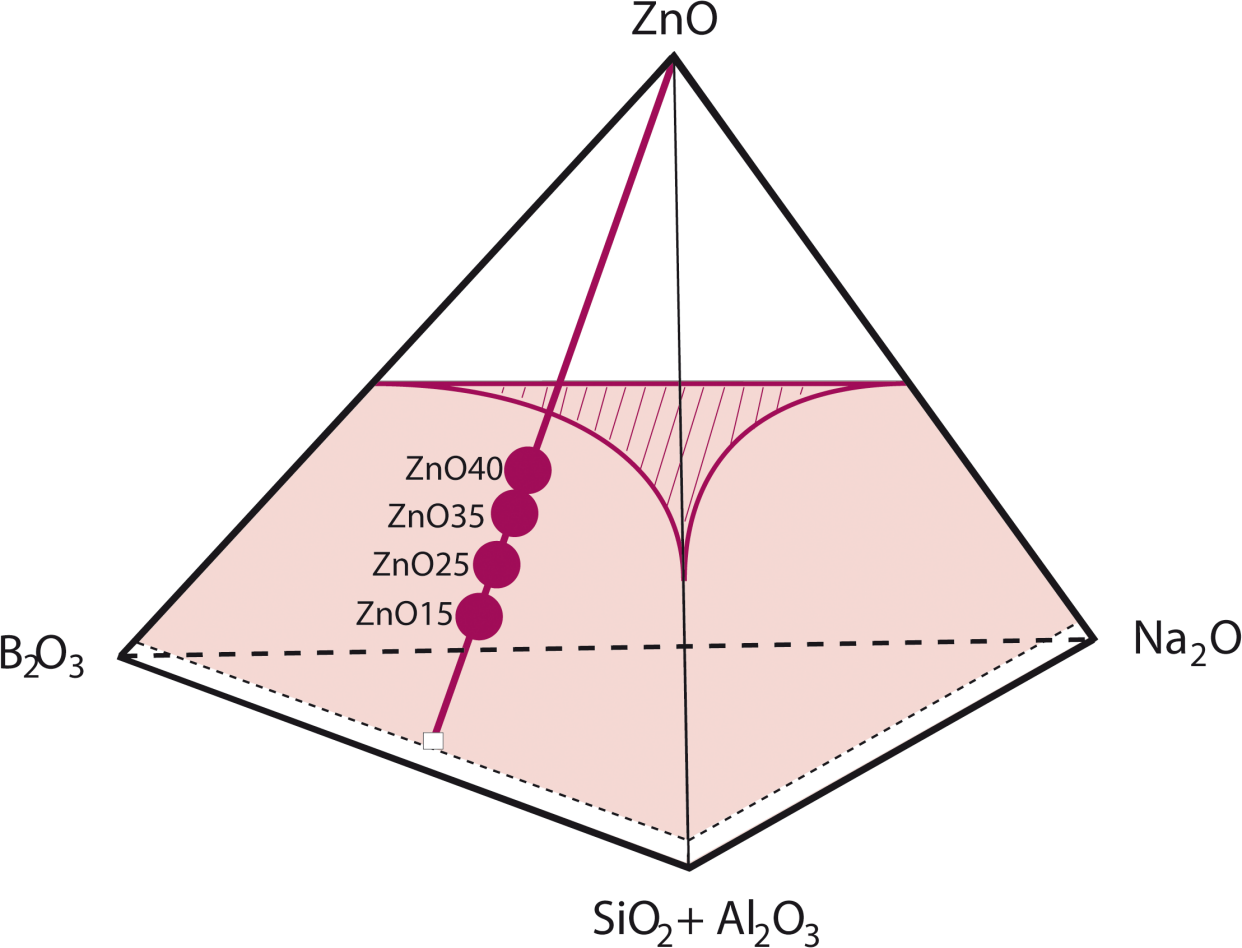
Tentative quaternary B_2_O_3_-(SiO_2_+Al_2_O_3_)-Na_2_O-ZnO system at 1300°C showing the compositions studied as well as the miscibility volume.

A new family of non-toxic biocides based on low melting point (1250°C) transparent glasses with high content of ZnO (15–40wt%) belonging to the miscibility region of the B_2_O_3_-SiO_2_-Na_2_O-ZnO system (Fig 1) has been developed. The chemical composition is given in Table 1. The physical properties as transition temperature (T_g_), hemisphere temperature (T_h_) and thermal expansion coefficient (α) for the ZnO containing glasses are summarized in Table 2. The T_g_ was determined by DTA.

**Table 2:** Transition temperature (T_g_), hemisphere temperature (T_h_) and thermal expansion coefficient (α) of the ZnO15, ZnO25, ZnO35 and ZnO40 glass.

	T_g_ (°C)	T_h_ (°C)	α (10^-6^K^-1^)
**ZnO15**	545.0±0.1	764.0±0.1	7.74±0.10
**ZnO25**	530.0±0.1	727.0±0.1	8.33±0.10
**ZnO35**	510.0±0.1	666.0±0.1	10.70±0.10
**ZnO40**	560.0±0.1	965.0±0.1	7.45±0.10

**Fig 2:**
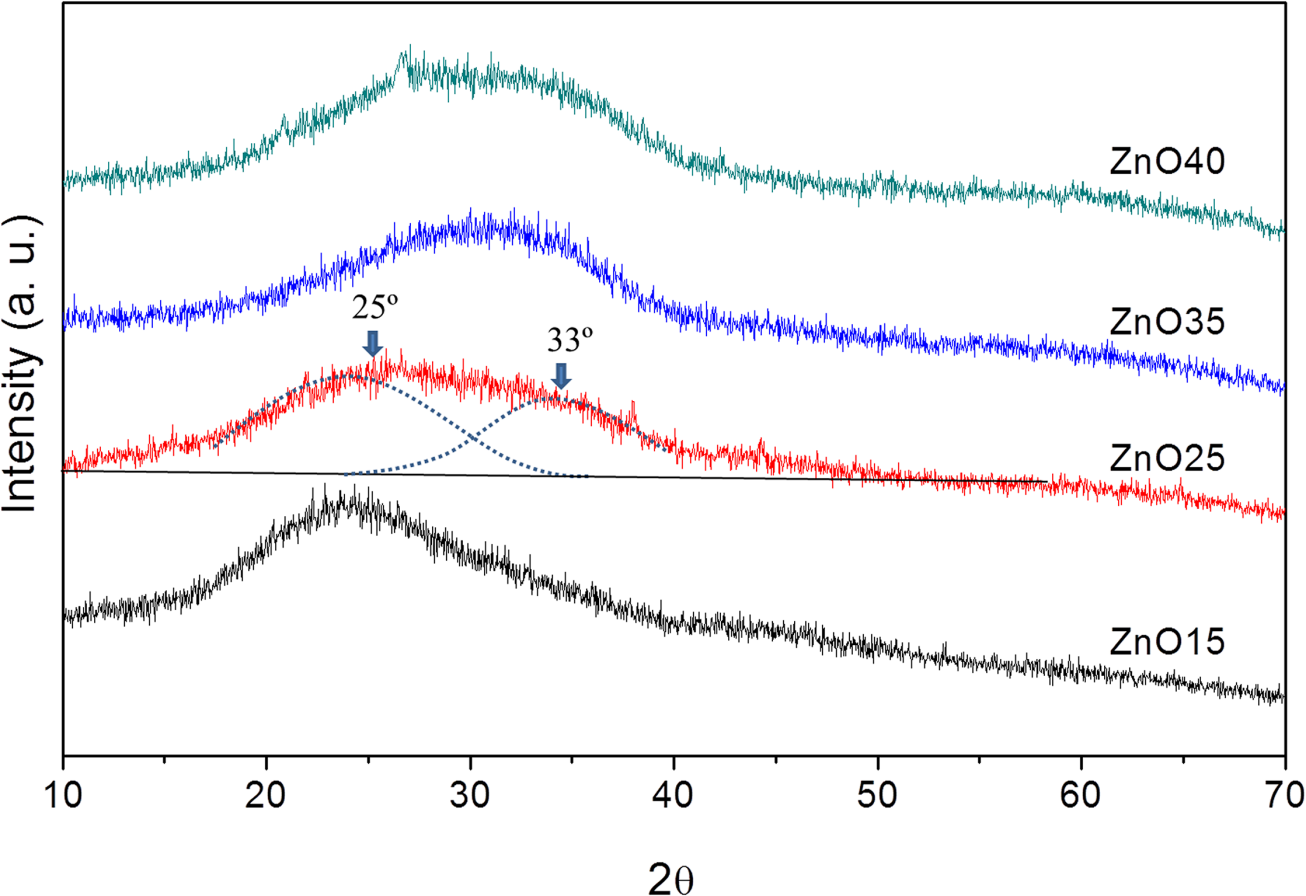
XRD patterns for the ZnO15, ZnO25, ZnO35 and ZnO40 glasses.

The XRD pattern for each glass is plotted in F. 2. In this figure the characteristic bell curve for amorphous substances can be seen. In this regard it is important to point out the following: (i) The XRD pattern of ZnO15 is the typical one of the silicon-borate glasses with an amorphous band with the maximum at 2θ∼23°;(ii) in the case of ZnO25 this amorphous band is composed of 2 components with the maximum at 2θ∼25° and 2θ∼33° (Fig 2). This is a clear indication of the existence of 2 glass-networks, this second one may be related to a Zn-reach network; and (iii) in the ZnO35 and ZnO40 XRD patterns the second band is predominant. These results are in good agreement with the NMR study.

**Fig 3:**
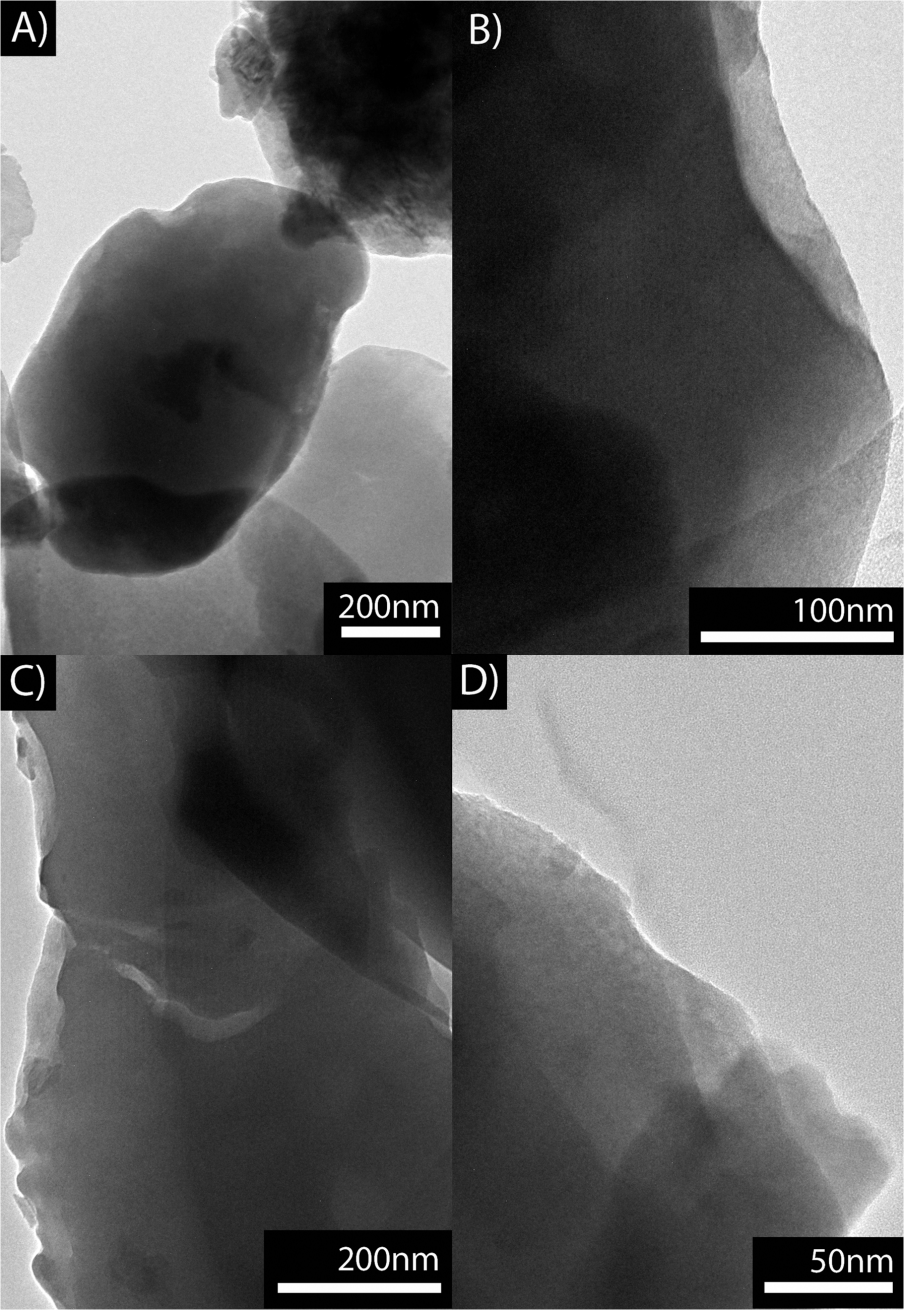
Transition electron micrographs of the glass powders: A) ZnO15, B) ZnO25, C) ZnO35 and D) ZnO40.

The transition electron micrographs of the glass powders ZnO15, ZnO25, ZnO35 and ZnO40 are shown in Fig 3. In the case of the ZnO15 glass, nanometric crystalline precipitates can be observed. It is important to point out that all the obtained glasses were found to be transparent without sign of phase separation (Fig 3). It means that this series of compositions are located inside the miscibility volume of the B_2_O_3_-SiO_2_-Na_2_O-ZnO simplified quaternary system (Fig 1).

### 3.2 Structure (NMR Study)

**Fig 4:**
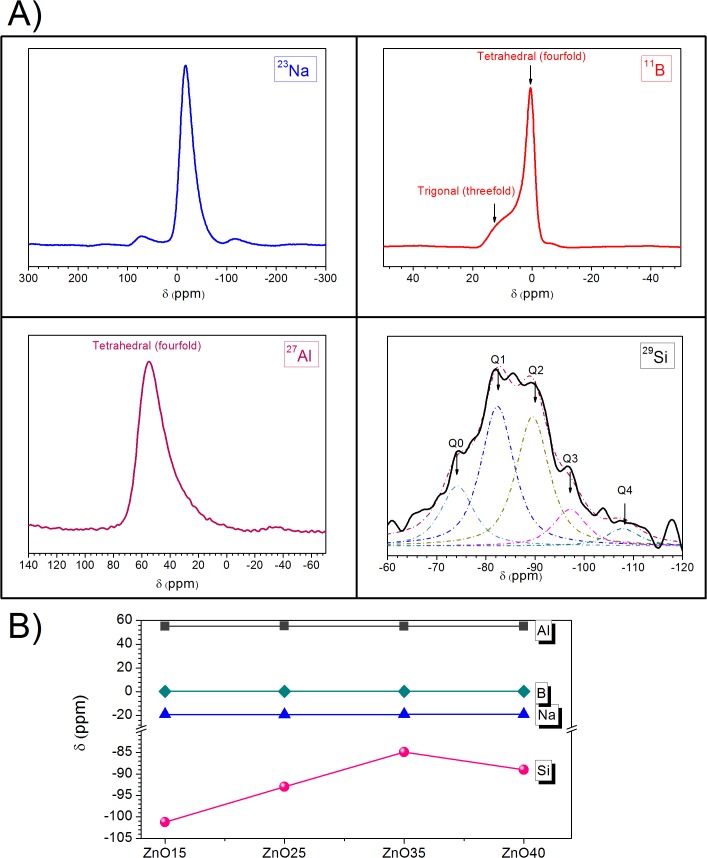
A) ^23^Na, ^11^B, ^27^Al, ^29^Si NMR spectra of the glass powder ZnO25. B) Chemical shift values as a function of zinc content in ZnO15, ZnO25, ZnO35 and ZnO40.

The local structure of glasses was assessed by NMR spectroscopy. For that the local environment of B, Na, Al and Si atoms was analyzed with the MAS-NMER technique. The ^11^B, ^23^Na, ^27^Al and ^29^Si, MAS-NMR spectra of the glass powder ZnO25 is displayed in Fig 4A as example. Chemical shift values as a function of Zn content in ZnO15, ZnO25, ZnO35 and ZnO40 are compiled in Fig 4B. This figure reveals that the addition of ZnO does not affect Na, Al and B NMR spectra, indicating that structural positions occupied by B, Al atoms (BO_4_, AlO_4_) do not appreciably change along the series but it does the Si (SiO_4_) one. The increment in the ZnO content causes the maximum shifting from the Si NMR envelope -100 ppm (ZnO15) to -85ppm (ZnO35) showing a linear tendency. Based on the chemical shift values reported in literature [46], the SiO_4_ tetrahedral in the ZnO15 and ZnO25 glasses is involved basically in Q^3^ environments but it changes to a Q^2^ one in the ZnO35 glass. Conversely, the ZnO40 glass breaks this tendency showing the spectral maximum at -90 ppm that corresponds again to Q^2^ environments. These structural changes affect the T_g_, T_h_ as well as the thermal expansion coefficients of the glasses as can be observed in Table 2. In the case of ZnO15 to ZnO35 there is a monotonically decrease of the fraction of silica (Table 1) and as a consequence, the T_g_ and T_h_ decrease and the thermal expansion coefficients increase (Table 2). Conversely, the ZnO40 glass displays a completely different trend, the T_g_ and T_h_ increase and the thermal expansion coefficient decreases (Table 2) showing a Q^2^ structure as the ZnO35 glass. This particular behavior can be rationalized considering a change in the structural role of ZnO from network modifier to network former along the series. In the case of ZnO15, ZnO25 and ZnO35 glasses it is clear that the ZnO acts as a modifier and produces the depolymerization of the SiO_2_. However, in the case of the ZnO40, where silicon Q^2^ associations prevail, the ZnO must act as network former diluting less polymerized silicon associations in resulting networks. This fact can also explain the results of the chemical durability study (Table 2).

### 3.3 Antimicrobial Activity

#### 3.3.1 Antimicrobial Activity

The logarithm reduction was used to characterize the biocide activity of the glass powders (Eq 3):

Logarithmreduction=logA−logB (3)

where A is the average number of viable cells from inoculum control (microorganisms without biocide agent), and B is the average number of viable cells from the culture with the glass powder after 24h.

**Fig 5:**
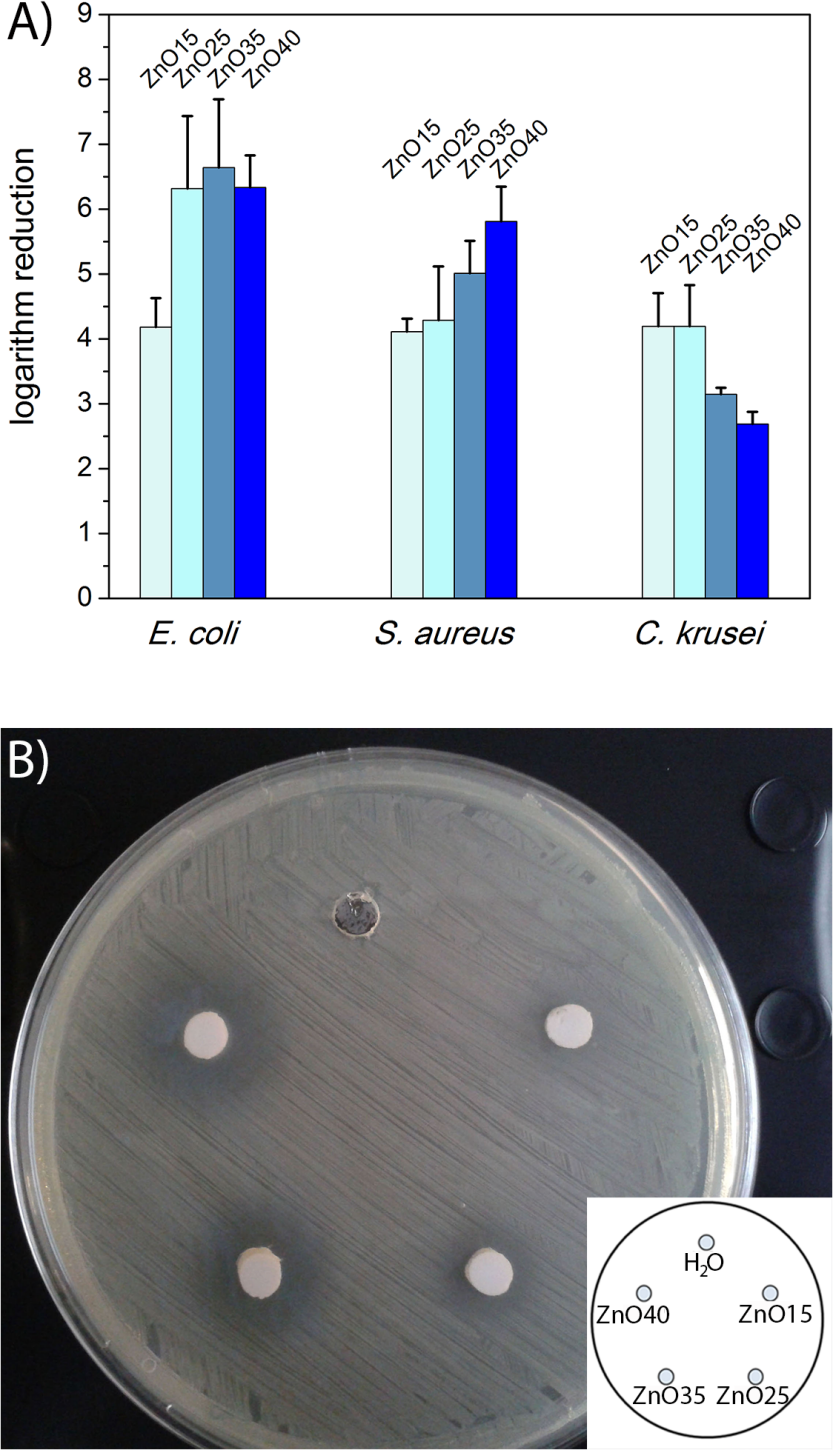
A) The logarithm reduction for the glass powders ZnO15, ZnO25, ZnO35 and ZnO40 at 5mg/ml against *E*. *coli*, *S*. *aureus* and *C*. *krusei* after 24h and B) Zone of inhibition test for distillate water, ZnO15, ZnO25, ZnO35 and ZnO40 glasses.

The logarithm reductions for the glass powders ZnO15, ZnO25, ZnO35 and ZnO40 against *E*. *coli*, *S*. *aureus and C*. *krusei* after 24h with a concentration of 5mg/ml in the cultures are represented in Fig 5A. A significant logarithm reduction (>3log) against *E*. *coli*, *S*. *aureus* and *C*. *krusei* for all glasses was obtained. A logarithm reduction higher than 3 means a safe disinfection and a high antimicrobial activity [47].

The biocidal activity for the glass powders increases with the increase in ZnO concentration for both *E*. *coli* and *S*. *aureus*. However, an opposite trend is observed for *C*. *krusei*. In previous work [40] it has been proven that two antibacterial mechanisms can operate in a glass powder: (i) the release of Zn^2+^ from the glass particle surface (this one is clearly the most important one in this series of ZnO containing glasses) and (ii) the glass particles can also be attached by electrostatic forces to the microorganism membrane, then the Zn^2+^ release at the interface could trigger the distortion of the membranes electrochemical potential, altering membrane transport of ions and/or nutrients causing the cell death. This mechanism is much depending on the nature of the membranes as well as the structure of the glass particles. In the case of bacteria the model described by Padmavathi et al. [48] claims that the ZnO can be activated in the presence of visible and/or ultraviolet light generating ROS (OH^-^, H_2_O_2_ and O_2_^2-^). The hydroxyls and superoxides cannot penetrate into the cell and they are caught in the external membrane, but the H_2_O_2_ penetrates causing oxidative stress and the death of the cell. Xie et al. [49] suggest a similar model in which the ZnO has a direct effect on the membrane modifying its permeability which increases the ZnO concentration inside of the cell where it induces oxidative stress. There studies state that Zn^2+^ directly induces a significant increment in reactive oxygen species (ROS) intracellular due to the Zn^2+^ interacts with the thiol-groups of the enzymes in charge of the breathing cycle of the bacteria (autoxidation of the NADH dehydrogenase II). The case of the yeast is different because these microorganisms are eukaryotic, therefore they have mitochondria. Following the model of Li et al. [50] the increase in the intracellular concentration of Zn^2+^ disrupts the mitochondria’s function and produces its collapse releasing ROS which induces oxidative stress. Finally, the membrane is broken and the components that produce the apoptosis are released. Besides, following Fronher et al. [51]*C*. *krusei* has a defense system against oxidation due to the fact that it presents at least 6 gens which codify putative enzymes of superoxide dismutase (bacteria present one or two). Four of these enzymes are Cu-Zn dependent and some of them are located in the membrane and they are only found in Candida. This fact can explain the observed behavior in the case of the yeast. More research would be required in the future for a complete understanding of this phenomenon.

The antimicrobial activity displayed by the ZnO containing glasses was found to be similar to the one obtained for glasses containing silver and copper nanoparticles reported in the literature [52].

#### 3.3.2 Minimum Inhibitory Concentration (MIC) Determination

The minimum inhibitory concentration was also determined and was found to be 1.69±0.18mg/ml for ZnO15 glass, 1.67±0.15mg/ml for ZnO25 glass; 1.20±0.11mg/ml for ZnO35 glass and 0.82±0.17 for ZnO40 glass.

#### 3.3.3 Antibiograms

Fig 5B illustrates the antibacterial activity of ZnO containing glasses against *E coli*. Since *E*. *coli* is susceptible to ZnO, then a clear zone (zone of inhibition) appears on the agar plate around the wells loaded with glasses suspensions, indicating that bacteria are not growing in this area. The zone of inhibition test is designed to assess the antimicrobial activity of those antimicrobial agents in which the activity is directly dependent on the leachability of the antimicrobial agent. The size of the zone of inhibition is usually related to the level of antimicrobial activity. Therefore the presence of a zone of inhibition indicates that ZnO is leaching, that it is able to diffuse into the agar and is effective against bacteria. The size of the zone of inhibition is related to the level of antimicrobial activity but also to the diffusion constant for the antimicrobial agent in the media and to the total amount of agent available to diffuse [53]. Thus, the correlation between the diameter of the zone of inhibition and the ZnO content in the glasses would suggest that ZnO40 is the most potent glass followed by ZnO35, ZnO25 and ZnO15. As shown in Fig 5B the largest diameter of the visible zone of inhibition is presented by ZnO40, followed by ZnO35, ZnO25 and ZnO15 which is in correlation with their ZnO content.

#### 3.3.4 Presence of Antimicrobial Leaching

The procedure for determining the presence of antimicrobial leaching was designed to ascertain whether or not the antimicrobial activity of our glasses is dependent upon direct contact of microbes with glass particles (as in the case of Ca rich containing glasses [54,55]) or on the contrary if it is due to the release of Zn^2+^ to the medium. The absence of turbidity in the tubes containing the ZnO-conditioned medium means bacteria are not growing. Results clearly indicate that the ZnO released from the glass is responsible of the observed biocide activity and the contact with glass particles is not necessary to inhibit the growth of *E*. *coli*. These results are in a good agreement with the antibiograms results. This mechanism enhanced the range of applications for these particular ZnO containing glasses. The corresponding glasses without ZnO tested as controls produced no effect on the viability of the microorganisms.

### 3.4 Biocompatibility Test

The cytotoxicity of the zinc containing glasses was evaluated using the NIH-3T cells. A viability >80% which means no cytotoxicity was obtained for a glass concentration of 5mg/ml.

### 3.5 Chemical Durability

The normalized elemental mass release (NR_e_), for Na, B, Si, Al and Zn calculated from the PCT together with two regulations for the solubility of borosilicate glasses used as containers of nuclear wastes, (i) the US Department of Energy (DOE) limit for Handford low-activity waste (LAW) borosilicate glass [56] and (ii) Defense Waste Processing Facility-Environmental assessment (DWPF-EA) glass [57] are compiled in Table 3. The NR_Na_ and NR_Si_ for all glasses were below or close to the EA limit but in the case of the NR_B_ the ZnO15 glass was over the EA limit, therefore this glass is the least chemically stable while the ZnO40 glass is also below the DOE limit, showing the highest chemical durability. This indicates that the addition of ZnO >35% enhances its resistance against chemical attacks. Probably, the ZnO fraction incorporated as network formed stabilizes the glass network.

**Table 3:** Normalized mass release (g/m^2^) from PCT for the ZnO containing glasses.

	NR_Na_	NR_B_	NR_Si_	NR_Al_	NR_Zn_
**ZnO15**	6.421±0.001	12.280±0.001	0.189±0.001	0.021±0.001	0.002±0.001
**ZnO25**	3.176±0.001	5.418±0.001	0.056±0.001	0.000±0.001	0.001±0.001
**ZnO35**	4.335±0.001	3.888±0.001	0.013±0.001	0.001±0.001	0.001±0.001
**ZnO40**	0.403±0.001	1.028±0.001	0.011±0.001	0.002±0.001	0.001±0.001
**DOE limit**	2	2	2	-	-
**EA limit**	6.67	8.35	1.96	-	-

### 3.6 Lixiviation of Zn^2+^

The Zn^2+^ released from the glasses to the media was measured in the PCT and in different media (distilled water, distilled water with 3wt% NaCl (sea-like water), LB and DMEN medium). The release of Zn^2+^ (ppm) from the zinc containing glasses (5mg/ml) in distilled water after 14 days is shown in Table 4. The release of Zn^2+^ (ppm) from ZnO15 and ZnO35 after 1 day in distilled water with 3% NaCl, LB medium and DMEN medium is collected in Table 5. The pH is also included in Tables 4 and 5.

**Table 4:** Zn^2+^ (ppm) lixiviated from the zinc containing glasses in distilled water (5mg/ml) and pH.

		1 day	4days	6days	14days
**ZnO15**	**Zn** ^**2+**^ **(ppm)**	0.4000±0.0050	0.4000±0.0067	0.5000±0.0017	0.6000±0.0017
	**pH**	7.3±0.1	8.0±0.1	8.3±0.1	8.4±0.1
**ZnO25**	**Zn** ^**2+**^ **(ppm)**	0.2000±0.0071	0.4000±0.0096	0.4000±0.0011	0.5000±0.0010
	**pH**	7.4±0.1	8.1±0.1	8.5±0.1	8.4±0.1
**ZnO35**	**Zn** ^**2+**^ **(ppm)**	0.5000±0.0016	0.8000±0.0037	1.0000±0.0050	1.1000±0.0040
	**pH**	7.4±0.1	7.5±0.1	7.5±0.1	7.8±0.1
**ZnO40**	**Zn** ^**2+**^ **(ppm)**	1.8000±0.0020	2.3000±0.0036	3.7000±0.0030	4.2000±0.0040
	**pH**	7.1±0.1	7.3±0.1	7.5±0.1	7.3±0.1

**Table 5:** Zn^2+^ (ppm) lixiviated from the zinc containing glasses in distilled water with 3% NaCl, LB and DMEN (5mg/ml) after 1 day. The pH is also shown.

	ZnO15		ZnO35	
	Zn^2+^(ppm)	pH	Zn^2+^(ppm)	pH
**Distilled water 3wt% NaCl**	0.12±0.01	8.3±0.1	0.31±0.01	9.0±0.1
**LB**	100±0.012	7.8±0.1	126.3±0.16	8.5±0.1
**DMEN**	33.600±0.018	7.5±0.1	35.600±0.019	7.9±0.1

In the case of the PCT the level of Zn^2+^ in the leachate was found to be <5ppm, below the toxic level for drinking water (15ppm) [58]. The Zn^2+^ released from the glass powders in the distilled water was found to be ~0.2–4ppm (Table 4). The Zn^2+^ lixiviated from the glasses ZnO15, ZnO25 and ZnO35 is <1ppm. This is the toxic limit for blue algae [59] which are considered one of the most sensitive organisms to heavy metals. The release of Zn^2+^ in distilled water with 3wt% NaCl, which is the concentration of salt in the sea, is <15ppm that is the toxic level for the drinking water. The possible toxicity in the other media can be solved decreasing the concentration of the powder. The difference in the releasing of the zinc is due to the ions and amino acids contained in media. Further studies will be developed in order to establish the exact component that causes this increment in the release of the zinc.

## Conclusions

In summary, these new family of glasses containing a high fraction of ZnO (>15wt%) can be considered chemically stable, non-toxic and with an excellent activity (>3 log reduction) against *E*. *coli* (Gram-), *S*. *aureus* (Gram+) and *C*. *krusei* (yeast). This series of glasses at the concentration of 2mg/ml was found to be at the same time biocompatible with NIH-3T3 cells (cell viability >80%). Because of this, those glasses can be used as non-toxic and green antimicrobial agents in a wide range of potential applications: e.g. as antifouling agents, surgical equipment, protective apparels in hospitals, water purifications systems, food packaging, food storages or textiles.

## Supporting Information

S1 Fig Determination of the MIC for ZnO15, ZnO25, ZnO35 and ZnO40 glasses. Replicate 1.
(TIF)

S2 Fig Determination of the MIC for ZnO15, ZnO25, ZnO35 and ZnO40 glasses. Replicate 2.
(TIF)

S3 Fig Determination of the MIC for ZnO15, ZnO25, ZnO35 and ZnO40 glasses. Replicate 3.
(TIF)
