# Correction: Antibacterial and Antifungal Activity of ZnO Containing Glasses

**DOI:** 10.1371/journal.pone.0136490

**Published:** 2015-08-21

**Authors:** The PLOS ONE Staff

## Notice of Republication

This article was republished on August 7, 2015, to replace Fig 2, which was incorrect. The publisher apologizes for the error. Please download this article again to view the correct version.
