# Supplementary figures and images for: Antibacterial and Antifungal Activity of ZnO Containing Glasses
Source: PLoS One. 2015 Jul 31;10(7):e0132709. doi: 10.1371/journal.pone.0132709 (PMC4521915; doi:10.1371/journal.pone.0132709)

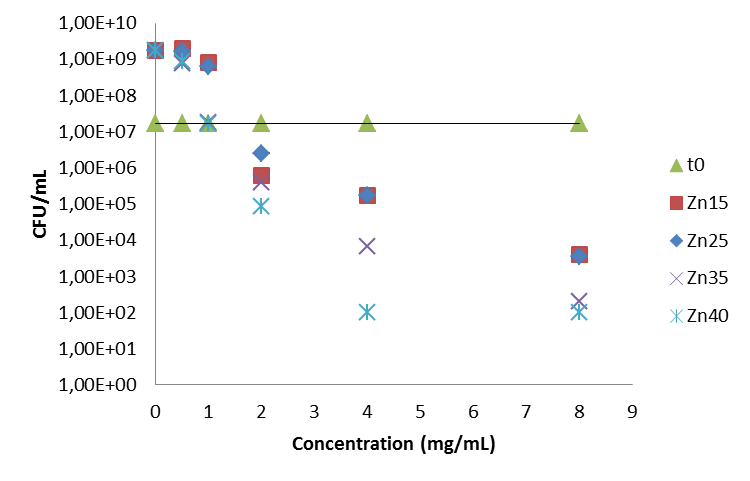

Supplement: S1 Fig — Replicate 1. (TIF) [file pone.0132709.s001.tif]

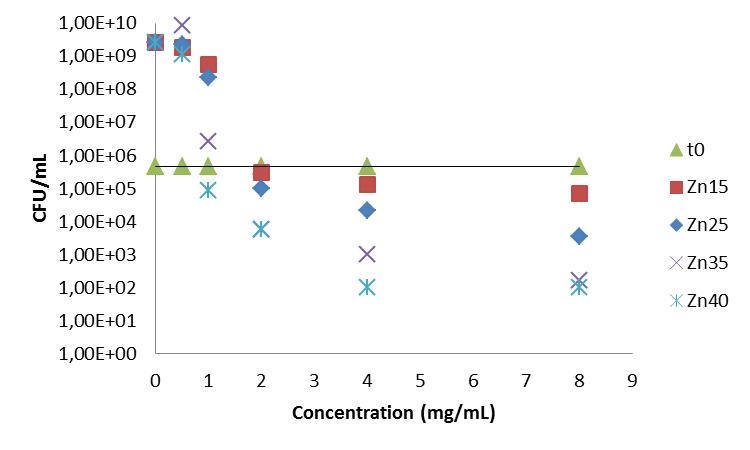

Supplement: S2 Fig — Replicate 2. (TIF) [file pone.0132709.s002.tif]

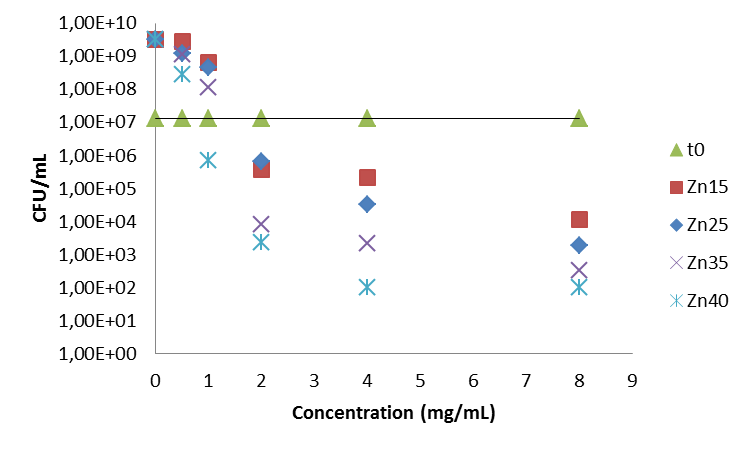

Supplement: S3 Fig — Replicate 3. (TIF) [file pone.0132709.s003.tif]
